# Presentations Related to Acute Paracetamol Intoxication in an Urban Emergency Department in Switzerland

**DOI:** 10.1155/2019/3130843

**Published:** 2019-12-06

**Authors:** Natalia Piotrowska, Jolanta Klukowska-Rötzler, Beat Lehmann, Gert Krummrey, Manuel Haschke, Aristomenis K. Exadaktylos, Evangelia Liakoni

**Affiliations:** ^1^Department of Emergency Medicine, Inselspital, University Hospital Bern, University of Bern, Bern, Switzerland; ^2^Clinical Pharmacology and Toxicology, Department of General Internal Medicine, Inselspital, Bern University Hospital, University of Bern, Bern, Switzerland; ^3^Institute of Pharmacology, University of Bern, Bern, Switzerland

## Abstract

**Aim:**

To investigate the characteristics of Emergency Department (ED) presentations due to acute paracetamol intoxication.

**Methods:**

Retrospective observational study of patients presenting to the ED of Bern University Hospital between May 1, 2012, and October 31, 2018, due to a paracetamol overdose (defined as intake of >4 g/24 h). Cases were identified using the full-text search of the electronic patient database and were grouped into intentional (suicidal/parasuicidal) and unintentional intoxications (e.g., patient unaware of maximal daily dose).

**Results:**

During the study period, 181 cases were included and 143 (79%) of those were intentional. Compared to the patients in the unintentional group, patients in the intentional group were more often female (85% vs 45%, *p* < 0.001) and younger (median age 23.0 vs 43.5 years, *p* < 0.001), more frequently suffered from psychiatric comorbidities (93%, (including 49% with borderline personality disorder) vs 24%, *p* < 0.001), and paracetamol was more often taken as a single dose (80% vs 13%, *p* < 0.001). Although the median daily ingested dose was lower in the unintentional than in the intentional group (8.2 g vs 12.9 g, *p* < 0.001), patients in the unintentional group presented later (29% vs 84% within 24 h of ingestion, *p* < 0.001), included more cases of acute liver failure (nine (24%) vs six (4%), *p* < 0.001), and were more often hospitalised (24% vs 52% treated as outpatients, *p*=0.002). There were no significant differences between the groups regarding drug-induced liver injury (seven cases (5%) in the intentional and one (3%) in the unintentional group) or fatalities (one in each group).

**Conclusions:**

The majority of presentations due to paracetamol poisoning were intentional, most commonly in female patients with borderline personality disorder. Patients with unintentional paracetamol intoxication had worse outcomes with respect to acute liver failure and hospitalisation. Future preventive measures should raise awareness of paracetamol toxicity in the general population and encourage particular attention and frequent follow-ups when prescribing paracetamol for vulnerable groups.

## 1. Introduction

Paracetamol (also known as acetaminophen, N-acetyl-*p*-aminophenol, and abbreviated as APAP) is a centrally acting analgesic and antipyretic agent with minimal anti-inflammatory properties [1]. It was first introduced to the market in the 1950s [2] and has since become the most commonly used drug worldwide for the treatment of pain and fever [2, 3]. Paracetamol is often the agent of choice in children, due to the well-known adverse effects of alternatives (e.g., Reye syndrome after intake of aspirin [4]). Paracetamol may be associated with neurodevelopmental and behavioural disorders such as attention deficit hyperactivity disorder (ADHD), and there are ongoing studies on whether it is linked to an increase in the incidence of asthma in children [3]. Nevertheless, paracetamol has been the recommended analgesic drug during pregnancy and breastfeeding for decades [2]. In most countries, paracetamol is available without a prescription as an over-the-counter (OTC) drug and is generally considered safe if used at the recommended dosage (i.e., ≤4 g per day in adults [5]).

The first reports of adverse hepatotoxic effects and fatal outcomes after paracetamol overdose can be found even in the 1970s [6]. Paracetamol's hepatotoxicity is generally considered to be associated with its highly reactive metabolite N-acetyl-*para*-benzoquinone imine (NAPQI), which can lead to hepatocyte damage [5]. Reported risk factors for the development of hepatotoxicity include chronic alcohol ingestion, chronic malnutrition, advanced age, genetic factors, and comedication with drugs that induce hepatic cytochrome P450 (CYP) [5, 6]. The management of paracetamol poisoning depends on the mode of intake. After a single ingestion, paracetamol concentration in serum (performed at least four hours after ingestion, i.e., after complete absorption of the drug) can be evaluated using the Rumack–Matthew nomogram, and the decision about therapy with N-acetylcysteine (NAC) is then based on the nomogram's treatment line [7]. However, this nomogram cannot be used after staggered intakes of paracetamol in overdose [8].

Beside unintentional overdoses, paracetamol is often involved in suicidal attempts. In the United States of America (USA), intentional overdoses, i.e., suicidal/parasuicidal behaviour, contribute to about 74–92% of all paracetamol overdoses [9]. In the United Kingdom (UK), the corresponding figure is ca. 75% [10, 11]. Although fatalities associated with paracetamol are rather rare in Switzerland [6], the number of intoxications caused by paracetamol overdoses increased more than twofold between 1995 and 2016 [12], and over 50% of those cases were intentional (suicidal) [6].

The present study was planned to establish the prevalence, patterns, and susceptible groups of paracetamol intoxications. It covers a period of six and a half years and describes the frequency, characteristics, and management of presentations related to paracetamol intoxication at an Emergency Department (ED) in Bern, Switzerland. The results could have major implications for epidemiology and public health.

## 2. Materials and Methods

This retrospective observational study included all patients ≥16 years of age presenting to the ED of the University Hospital Bern between May 1, 2012, and October 31, 2018, due to paracetamol overdose (defined as intake of >4 g/24 h). The ED of Bern University Hospital is both a primary care facility (walk-in patients) and tertiary referral center for hospitals in the greater Bern area, with about 48000 emergency admissions a year (2018). The study was approved by the local ethics committees (No. BE 2018-02275).

Cases were identified by using a search function of our electronic ED patient database E.care (E.care BVBA, ED 2.1.3.0, Turnhout, Belgium). This medical database allows recall of past diagnostic reports, consultations, and other relevant medical documents. A full-text search was performed with “paracetamol” as search term in the diagnosis field of the ED report. The retrieved cases were then reviewed, and the cases were selected where the reason of presentation was (suspected) paracetamol intoxication. Cases were then excluded if the patient had not given general consent to process his or hers medical data for research purposes. In the final step, we selected only those patients with paracetamol overdose defined as intake of more than 4 g paracetamol in 24 h, as based on information given by patients, patient's families/friends, or paramedics. We also included cases in which no exact information was available about the ingested dose, but the paracetamol level in serum was above the therapeutic concentration, defined as a maximum of 32 *μ*g/mL (= 212 *μ*mol/L) [6, 13]. We excluded cases with no available information about the ingested dose and serum paracetamol level not above the therapeutic concentrations. For patients presenting to the ED more than once due to paracetamol intoxication during the study period, each individual presentation was considered one case.

The following parameters were extracted (if available) from the charts of the included patients: age, sex, nationality, circumstances of exposure (i.e., accidental or intentional/suicide), paracetamol dose, paracetamol level in serum, date of presentation, reasons for intake, simultaneous intake of other substances, mode of intake (single vs. increased intake over several days), latency from first intake to presentation, risk factors, psychiatric comorbidities, laboratory findings, treatment provided, type of discharge from the ED (e.g., inpatient admission and outpatient therapy), and outcome (e.g., complications and death).

In terms of outcome, drug-induced liver injury (DILI) was defined as an elevation of alanine aminotransferase (ALT) over 5*x* the upper limit of normal (i.e., >175 IU/L), or elevation of alkaline phosphatase (ALP) over 2*x* the upper limit of normal (i.e., >210 IU/L), or elevation of ALT over 3*x* the upper limit of normal (i.e., >105 IU/L) and simultaneous elevation of bilirubin over 2*x* the upper limit of normal (i.e., >34 *μ*mol/L) [14]. Acute liver failure (ALF) as an extreme form of DILI was defined as elevation of transaminases over 10*x* the upper limit of normal (i.e., >350 IU/L) with elevation of INR over 1.5 and an altered level of mental status developing in less than 26 weeks in patients without preexisting liver disease [15, 16].

Cases were grouped into intentional (i.e., suicidal or parasuicidal attempts) and unintentional (e.g., the patients did not know or ignored the maximal allowed daily dose because of lack of understanding of overdose-related risks). Differences between the two groups were tested using the chi-squared or Fisher's exact test for categorical variables, the *t* test for normally distributed continuous variables, and the Mann–Whitney test for nonparametric variables (*p* < 0.05 was considered statistically significant). Statistical analyses were conducted using SPSS statistical software (IBM SPSS Statistics 25.0).

## 3. Results

A total of 987 potential cases with the word “paracetamol” in the diagnosis field of the ED were retrieved in the first step of our search. From this group, 214 cases were selected in which the reason for current presentation was (suspected) paracetamol intoxication. Eighteen cases had to be excluded because of lack of general consent, and 15 further cases did not fulfill the inclusion criteria (e.g., paracetamol intake of <4 g/24 h, no available information about the ingested dose, and serum paracetamol level not above the therapeutic concentrations (six cases)) and were also excluded. One hundred and eighty-one (181) cases of paracetamol intoxication were included in the final analysis (among them five cases without exact information about the ingested dose available, but paracetamol level in serum above the therapeutic concentration), including 38 (21%) unintentional and 143 (79%) intentional overdoses. There were no cases of simultaneous use of different paracetamol preparations and only two cases involving a paracetamol combination product containing tramadol and paracetamol.

**Figure 1:**
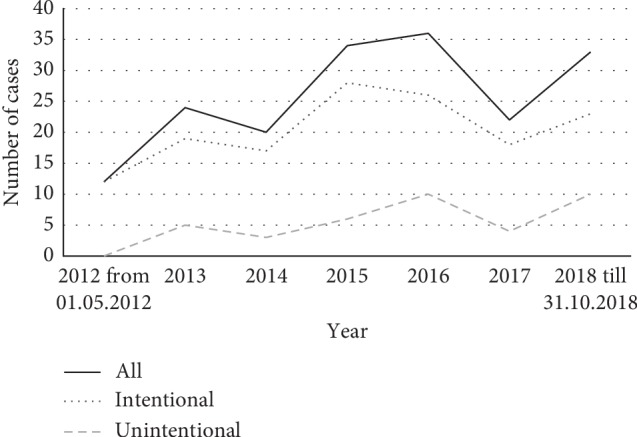
Annual distribution of presentations due to paracetamol intoxication (*N* = 181).

Figure 1 shows the annual distribution of paracetamol intoxication cases over the study period; intentional and unintentional cases are illustrated separately in addition to all cases.

An overview of the main characteristics of the paracetamol intoxication cases is provided in Table 1.

### 3.1. Intentional Paracetamol Intoxication (*n* = 143)

Among the 143 cases of intentional paracetamol overdoses, the median age was 23.0 (range 16–85) years, with a predominance of women (*n* = 121, 85%), and in 17% of the cases (*n* = 24), the nationality of the patients was not Swiss (Table 1). Most patients in this group had suicidal intention (134 cases, 94%), while nine (6%) reported no strict suicidal intention but intention to harm themselves by taking a paracetamol overdose. The median daily paracetamol dose ingested was 12.9 g; the maximal paracetamol dose 90 g (Table 1). In five cases, the dose of paracetamol was not known. The most common mode of paracetamol intake was ingestion as a single dose (80%, 115 cases) (Table 1); in 19 cases (13%), paracetamol intake was staggered, and in nine cases (6%), the mode of intake was not known. Most patients (120 cases, 84%) presented to the ED during the first 24 h after first paracetamol ingestion (Table 1). Twenty-two (22) patients (15%) presented more than 24 h after the first ingestion (longest interval between first ingestion and presentation: four days), and in one case, the time from ingestion to presentation was unknown. In 50% of the intentional cases (*n* = 72), other substances were taken together with paracetamol. The most common agents in those cases were psychotropic drugs (35 cases) and other analgesics (32 cases), followed by alcohol (19 cases). There were 32 cases with ingestion of three or more different substances. In 102 cases (71%), treatment included i.v. NAC, combined with activated charcoal in 23 (16%) cases with early presentation. Three patients (2%) were treated solely with charcoal, and 15 patients (11%) received no medical therapy. In 52% of the cases (*n* = 75), the patients were treated as outpatients; 6% (9 cases) were treated in a normal hospital unit, and 59 patients (41%) needed an intensive or intermediate care unit. Psychiatric comorbidities were known/documented in 133 cases (93%) (Table 1). Of these, the most common was borderline personality disorder (65 cases; 49%), followed by depressive disorder (44 cases; 33%). Among the borderline patients, four female patients presented more than four times due to paracetamol intoxication during the study period (max. number of presentations of the same patient: 12 times). Other psychiatric comorbidities were acute psychological crisis/depressive adjustment disorder (15 cases, 11%), schizophrenia (5 cases; 4%), addiction disease (2 cases; 2%), and single cases of patients with eating disorder and dementia. There were six cases of ALF in this group (4%): two in patients with chronic alcohol intake, one in a patient with malnutrition, and three without known risk factors. The median paracetamol dose taken by patients with ALF was 26 g (range 21–80 g). There were also seven cases of DILI (5%): two with malnutrition as a risk factor and five without risk factors. The median paracetamol dose taken by patients with DILI in this group was 35 g (range 15–90 g). The paracetamol level in serum was available in 137 (96%) of the intentional intoxication cases (exact time of measurement in most cases not known). Among these, the concentration was in the therapeutic range (i.e., <212 *μ*mol/L) in 39 cases and above the therapeutic range in 98 cases (median 476 *μ*mol/L, range 232–10236.6 *μ*mol/L). There was one fatal case of a 42-year-old female patient with a borderline personality disorder and intake of multiple drugs (metamizole, diclofenac, and ibuprofen), together with 24 g of paracetamol. NAC was started in this patient about five hours after paracetamol ingestion and was administered over 20 hours. Although the initially elevated liver enzymes recovered during hospitalisation, the patient died because of pneumonia and respiratory insufficiency after multiple ischemic strokes.

### 3.2. Unintentional Paracetamol Intoxication (*n* = 38)

Among the 38 cases of unintentional paracetamol intoxication, the median age was 43.5 (range 16–79) years; 45% of the patients were women (17 women; 21 men), and 26% (ten cases) were of non-Swiss nationality (Table 1). The most commonly reported reason for paracetamol intake was pain (35 cases, 92%), followed by common flu symptoms/fever (three cases, 8%). Patients with pain most commonly reported headache (13 cases), followed by abdominal pain (nine cases), toothache (six cases), local postoperative pain (three cases), and throat and back pain (two cases each). The maximal dose of ingested paracetamol was 25 g/24 h. The median dose per day was 8.2 g (range 5–25) (Table 1). In most cases (33 cases, 87%), the increased intake took place over many days because of insufficient pain relief and without respecting the maximum recommended daily dose, and most of the patients (27 cases, 71%) presented to the ED later than 24 h after first ingestion of paracetamol in overdose. The longest interval between first paracetamol ingestion and ED presentation was 21 days. In ten (26%) of the cases, the patients had also ingested substances other than paracetamol. The most commonly reported co-ingested substances in those cases were other analgesics (i.e., nonsteroidal anti-inflammatory drugs (NSAID) or metamizole) in six cases and alcohol (six cases). Psychiatric comorbidities were known/reported in 24% of the cases (*n* = 9). As regards treatment, most patients received i.v. NAC (30 cases, 79%). Due to the long latency from the first ingestion to presentation in most cases, activated charcoal was administered in only one of the cases receiving NAC. Seven patients (18%) who presented with normal liver enzymes and latency between presentation and paracetamol intake of more than one day received no treatment. Most patients were treated in a normal hospital unit (16 cases, 42%); 13 (34%) needed an intensive or intermediate care unit, and nine (24%) were treated as outpatients (Table 1). The most common psychiatric comorbidity was depressive disorder (four cases), followed by addiction disease (three cases) and borderline personality disorder (two cases). There were nine cases of ALF in this group (Table 1); six of them occurred in patients with preexisting risk factors (chronic alcohol intake (four cases), preexisting liver disease (one case) or malnutrition (one case)). In the ALF subgroup, the median dose of paracetamol was 8 g per day (range 5.5–15 g); however, the intake was commonly repeated over many days (eight of the nine patients with ALF). There was one case of DILI after intake of 15 g paracetamol as a single dose in a patient without risk factors and one fatal case due to progressive liver failure in a 64-year-old male patient with preexisting alcoholic liver cirrhosis, who consumed 9 g paracetamol in 24 h (NAC administered five days after paracetamol intake in this case). The paracetamol level in serum was measured in 30 (79%) of the cases of unintentional intoxication (exact time of measurement not documented in most cases). Among these, the concentration was in the therapeutic range (i.e., <212 *μ*mol/L) in 21 cases and above the therapeutic range in 9 cases (median 682 *μ*mol/L, range 330–1723.6 *μ*mol/L).

## 4. Discussion

In this retrospective study, at a large ED in Switzerland, there were approximately two presentations/month due to acute paracetamol overdose during the six and a half years investigated, with intentional intoxications forming the large majority (four out of five cases). Compared to the unintentional group, patients with intentional overdose were more often female, consumed higher single doses of paracetamol, and had more psychiatric comorbidities. Patients in the unintentional group were significantly older than the intentional group, presented later to the ED, and although the dose consumed was lower, the increased intake was more commonly repeated over many days. The unintentional group was also hospitalised more often and for longer duration and had significantly more ALF cases than the intentional group, while no significant differences between the groups were found for DILI and fatal outcomes.

Most of our patients with intentional paracetamol poisoning were female, which is in line with data from the general suicide statistics from Switzerland, according to which most suicidal attempts in women are by poisoning [17]. Data available from Switzerland show the increasing share of analgesics—particularly paracetamol [12]—as drugs chosen in suicidal attempts at self-poisoning [18]. According to the report on suicide patterns in the Bern agglomeration, analgesics were the second most common drug class after antidepressants that was involved in suicidal attempts [18]. According to studies from other countries, paracetamol is one of the most common drugs used in self-harm attempts [19–21], particularly among females and persons under 25 years [20]. Our results also support the findings from the UK and USA where intentional paracetamol intoxications account for more than 75% of all paracetamol poisonings [9, 11].

These findings have major public health implications and raise the question about the need for prophylactic measures, such as restricting availability of paracetamol, although this can differ between countries. In Switzerland, paracetamol can be bought as an OTC drug, but is only available in pharmacies or drug stores. In OTC formulations, the maximal paracetamol dose per pill is 500 mg (750 mg in rectal suppositories) and the maximal paracetamol pack contains 10 g (12 g as suppositories) [22]. However, with a physician's prescription, larger amounts of paracetamol can be purchased. There is a clear link between availability of paracetamol and the rate at which it is used for suicide [19, 23]. Some studies suggest that the availability of paracetamol through nonpharmacy outlets rather than pack size is the main factor contributing to the number of paracetamol poisonings [23]. This is supported by the finding that regulations restricting the pack size of paracetamol available in a single purchase did not seem to be very effective in the long term in the UK [24]. However, factors other than pack size and availability might also affect the rate of suicide attempts, as also illustrated by the short-term increase in paracetamol overdoses after depicting such an event in an episode of a popular medical television drama [25]. This all tends to highlight the complexity of the underlying mechanisms and the difficulty of taking timely and appropriate measures. In contrast to data from the USA [26], overdoses involving opioid-paracetamol combination products in pain patients do not seem to be a common problem in our population, since there were only two cases involving a compound paracetamol-product (combination of paracetamol and tramadol) and no cases involving multiple products containing paracetamol.

Cases of unintentional paracetamol overdose were relatively rare in our study. However, this group had a significantly higher rate of adverse outcomes in the form of ALF than the intentional group. In the unintentional group, most patients had taken paracetamol over many days in supratherapeutic doses and the presentation to the ED was often delayed. Together with the greater age of these patients, this might have contributed to the worse outcomes. These findings correlate with other studies that found a worse prognosis of unintentional paracetamol poisonings compared to patients with a single dose intake [11]. This was due to mainly staggered intake over many days, postponed presentation, and late beginning of antidote therapy [27]. Those cases should be managed with particular care, since paracetamol serum level and dose ingested per day, which are the usual evaluation tools of paracetamol intoxication in the ED, are often much lower than with the intentional cases, and this might lead to underestimating the high risk of adverse outcomes. Therapeutic measures—such as very liberal NAC administration—could help improve treatment and outcome in this critical group with less clear management and worse outcome. As regards prophylactic measures, unintentional poisoning cases might be avoided if contact to a primary care doctor is established in order to be able to provide in depth information about paracetamol toxicity and to schedule follow-ups to adjust the analgesic therapy as required (most cases in this group were pain patients, who performed self-medication with paracetamol and used supratherapeutic doses due to insufficient pain relief). This highlights the important role of family doctors and general practitioners, since they can provide more adequate long-term outpatient care compared to the treatment in the ED setting.

Psychiatric comorbidities were common in our study population, particularly in the intentional group. The most common psychiatric condition was borderline personality disorder, followed by depressive disorder. According to a recent study [28], patients who overdose paracetamol (intentionally as well as unintentionally) display a higher rate of depression, mismanagement of problematic chronic pain, frequent substance abuse, and increased impulsivity compared to the general population. On the basis of our findings, particular attention should be given to borderline female patients, as they seem to be at high risk of representing due to paracetamol intoxication shortly after discharge or while being hospitalised in a psychiatric unit. Frequent and intensive follow-up in these cases could help to avoid reoccurrence of suicide attempts.

In our study, there were two deaths after paracetamol intoxication, of which only one was directly related to paracetamol overdose. There were no cases of liver damage requiring liver transplantation and cases of ALF were relatively rare, particularly in the intentional group. The absolute number of adverse outcomes as a result of paracetamol overdose is lower in Switzerland [6] than in countries where paracetamol is available in nonpharmacy outlets. Examples are the USA and UK, where 458 [9] and 100–200 [29] deaths, respectively, occur each year due to paracetamol-associated overdoses, and paracetamol poisoning is the leading etiology of acute liver failure [26]. On the other hand, numbers per population should also be taken into consideration for comparisons. According to reports from the Scottish Liver Transplantation Unit [30], approximately 50% of the patients admitted with paracetamol-induced acute liver injury between December 1992 and March 2001 developed ALF. However, since approximately 50% of the patients die at home or are not referred to the Liver Transplantation Unit, based on a population of 4.99 million, the authors assume an incidence of 0.84 per 100000 for Scotland in 2001 (21 admissions) [30]. Thus, next to restriction of paracetamol availability, presence of risk factors, late presentation and postponed begin of antidote therapy, demographic differences, and referral procedures can also affect the frequency of paracetamol-related adverse events such as ALF.

Limitations of our study include the retrospective design and reporting bias (especially in cases of psychiatric comorbidities). Furthermore, 18 cases could not be included due to the lack of a general consent form, and parameters such as psychiatric comorbidities might be overrepresented in the intentional group due to patients presenting more than once. Moreover, ten cases were admitted to another hospital. Although these patients were stable at the time of admission, no information about the long-term outcome was available, and data from one ED may not be representative for the whole country. The strengths of the study include the sensitive search and the manual review of all cases. To our knowledge, this is the first study that has investigated the characteristics and susceptible groups of acute paracetamol intoxication in ED presentations in this population and contributes data to guide public health measures and to improve patient treatment.

## 5. Conclusions

On the basis of the findings of our study, ED presentations due to paracetamol intoxication are not uncommon. The majority of the cases constitute intentional, i.e., suicidal and parasuicidal, poisonings, often in patients with psychiatric comorbidities (mainly borderline personality disorder and depression) and with a high recurrence rate among female borderline patients. Special attention should be given to patients after unintentional paracetamol intoxication, as they make up a vulnerable group with worse outcome in terms of complications such as ALF—probably due to prolonged intake and delayed presentation. Preventive measures should be considered in order to increase awareness of paracetamol toxicity in the general population.

## Figures and Tables

**Table 1:** Main characteristics of cases presenting due to paracetamol intoxication.

	All (*N* = 181)	Intentional (*n* = 143)	Unintentional (*n* = 38)	*p*
Age in years	25.0 (16–85)	23.0 (16–85)	43.5 (16–79)	**<0.001**
Women	138 (76)	121 (85)	17 (45)	**<0.001**
Swiss citizens	147 (81)	119 (83)	28 (74)	0.18
Paracetamol g/24 h	10.8 (5–90)	12.9 (5–90)	8.2 (5–25)	**<0.001**
Intake as a single dose	120 (66)	115 (80)	5 (13)	**<0.001**
<24 h between ingestion and presentation	131(72)	120 (84)	11 (29)	**<0.001**
Intake of paracetamol only	99 (55)	71 (50)	28 (74)	**0.008**
Psychiatric comorbidities	142 (78)	133 (93)	9 (24)	**<0.001**
DILI	8 (4)	7 (5)	1 (3)	0.54
ALF	15 (8)	6 (4)	9 (24)	**<0.001**
Death	2 (1)	1 (<1)	1 (3)	0.30
Treatment as outpatients	84 (46)	75 (52)	9 (24)	**0.002**
Time of hospitalisation in days^£^	2.0 (1–21)	2.0 (1–21)	2.0 (1–15)	**0.001**

^£^Time of hospitalisation not known in ten cases with admission to another hospital. Values are expressed as median (range) or *n* (%); bold numbers indicate significant differences (*p* < 0.05) between the intentional and unintentional groups.

## Data Availability

The descriptive data used to support the findings of this study are included within the article.
